# From mechanisms of heart failure to clinical heart success

**DOI:** 10.1242/dmm.050282

**Published:** 2023-06-05

**Authors:** Jeroen Bakkers, Milena Bellin, Julija Hmeljak, Ravi Karra

**Affiliations:** ^1^ Hubrecht Institute-KNAW and Utrecht University Medical Center, Utrecht 3584 CT, The Netherlands; ^2^ Department of Pediatric Cardiology, Division of Pediatrics, University Medical Center Utrecht, Utrecht 3584 EA, The Netherlands; ^3^ Department of Biology, University of Padova, Padova 35131, Italy; ^4^ Veneto Institute of Molecular Medicine, Padova 35129, Italy; ^5^ Department of Anatomy and Embryology, Leiden University Medical Center 2333, ZC, Leiden, The Netherlands; ^6^ The Company of Biologists, Bidder Building, Station Road, Histon, Cambridge CB24 9LF, UK; ^7^ Division of Cardiology, Department of Medicine, Duke University Medical Center, Durham, NC 27710, USA; ^8^ Department of Pathology, Duke University, Durham, NC 27710, USA

## Abstract

**Summary:** This Editorial introduces DMM's new Special Issue on ‘Moving heart failure to heart success’. The Guest Editors reflect on how articles in the issue advance the cardiac research field.

Heart failure is characterised by the heart's inability to pump blood effectively, leading to reduced oxygen and nutrient supply to the body's organs and tissues, and is the leading cause of hospitalisation and of deaths in men and women worldwide (Benjamin et al., 2019). Heart failure occurs when the heart is weakened or damaged, or when there is a problem with the heart's valves or rhythm, and can affect the left or right or both sides of the organ. The onset of heart failure can be acute (sudden) or chronic (developing gradually over time) and can affect individuals at various stages of their lives. Once essentially untreatable, contemporary management of heart failure can delay or prevent decompensation or even supplant end-stage disease through mechanical assist devices or organ transplantation. However, significant gaps remain, as treatment of heart failure, at best, results in disease remission and is lifelong. In end-stage disease, both transplantation and mechanical circulatory support are associated with significant morbidity. Thus, to truly move from heart failure to heart success, there remains a critical need to identify curative strategies through research approaches that fundamentally address the roots of failure. Our goal in shaping this Special Issue of Disease Models & Mechanisms (DMM) was to compile original Research and Review-type articles that investigate the genetic and biological mechanisms of heart failure and identify potential therapeutic strategies (Hooper and Hmeljak, 2022). In this Editorial, we discuss how the articles in this Special Issue are contributing to the field's progress towards heart success.

## Underlying mechanisms

A significant risk factor for heart failure is congenital heart defects, which are structural abnormalities present at birth. Congenital heart defects occur frequently, affecting 8 out of every 1000 newborns, and are the major cause of morbidity and mortality in infants and children (Hoffman and Kaplan, 2002). Although surgical interventions like the Norwood procedure have improved the life expectancy of affected children, these patients remain at high risk of developing heart failure and arrhythmias later in life. The causes of congenital heart defects are diverse and include genetic, environmental and unknown factors. Importantly, treatment approaches for ‘garden variety’ adult heart failure may not be applicable to failure that results from congenital heart disease. Thus, research into cardiac development and disease mechanisms is essential to better understand the underlying causes of congenital heart defects, particularly because these relationships are not always obvious. Underscoring this complexity, a Review from Christian Mosimann's group summarises how defects in lateral plate mesoderm development drive syndromic congenital defects of multiple organs, including the heart (Kocere et al., 2023), and a Research article from Lelièvre et al. (2023) analyses how mutations in endoglin that are linked to a rare vascular developmental disorder cause congestive heart failure.

**Figure:**
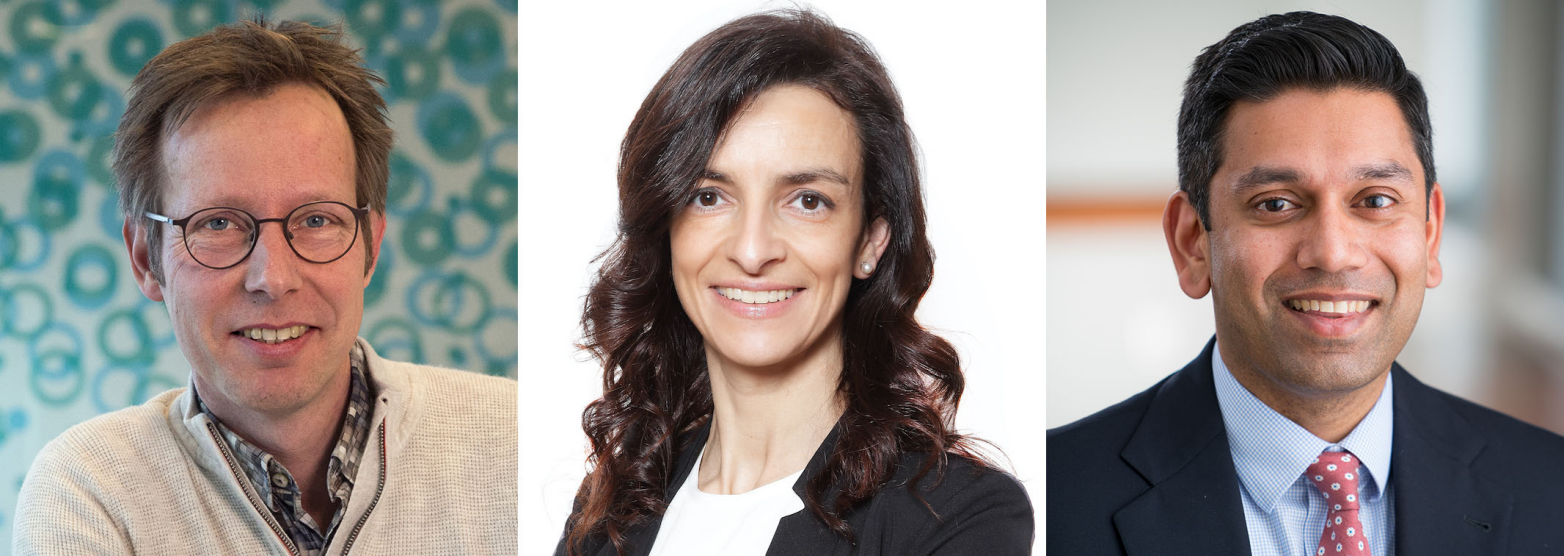
Jeroen Bakkers, Milena Bellin and Ravi Karra (left to right).

Beyond disordered heart development, arrhythmias, which occur due to disruptions in the heart's electrical system, represent another important risk factor for developing heart failure. Electrical currents that initiate contraction of cardiomyocytes can be disrupted by various factors, including structural remodelling of the heart, mis-regulation of ion channels, or a malfunctioning cardiac pacemaker. A Review by Vincent Christoffels and colleagues, published in this issue, explores the genetics of sinoatrial node dysfunction and how this contributes to arrhythmias (van der Maarel et al., 2023). New technologies, like spatially resolved and single-cell mRNA sequencing, have provided critical insights into the small population of pacemaker cells and offer the potential for gene therapy strategies and new treatments (Litviňuková et al., 2020; Wiesinger et al., 2022).

Cardiomyopathies are another group of underlying cardiac diseases that can lead to heart failure if left untreated. They are characterised by the remodelling of the heart muscle, which makes it more challenging to pump blood around the body. The heart muscle can become stretched in dilated cardiomyopathy, thickened in hypertrophic cardiomyopathy, or replaced by fibrotic and fat tissue in arrhythmogenic cardiomyopathy. Although several risk factors for cardiomyopathies are known, such as high blood pressure, abnormal heart rhythm and viral infections, there is growing evidence for genetically inherited traits that increase the risk of developing these conditions (Burke et al., 2016). To understand how genetics lead to cardiac remodelling in cardiomyopathies, accurate *in vitro* and *in vivo* models that faithfully recapitulate key aspects of these diseases are essential. Such models will enable the investigation into the underlying genetic mechanisms and the development of potential treatments for these conditions. Precise genome editing, as discussed in the Review from Eva Van Rooij's group (Kyriakopoulou et al., 2023), allows researchers to generate these much-needed models. Importantly, these approaches also offer tremendous potential for precise and personalised treatment of genetic cardiomyopathies (Kyriakopoulou et al., 2023).

Finally, acquired cardiomyopathies secondary to injury are a major cause of heart failure (Tsao et al., 2023). Coronary heart disease, manifesting as either acute myocardial infarction or chronic tissue ischaemia, can lead to cardiac muscle loss or dysfunction. Although less common, viruses, toxins or systemic inflammatory syndromes can similarly result in loss of tissue and heart failure. Not only do we need approaches to restore or replenish lost myocardium, but the community also needs model systems that accurately model these injuries to determine how unique injury environments might affect therapeutic responses.

## Developing, characterising and choosing the right models

In science, developing and choosing the right model is key for advancing knowledge in the field and this also holds true for heart failure. Because investigating the early stages of mammalian cardiac development is challenging due to the inaccessibility of the embryo *in utero*, researchers in the field are using a battery of models that are more accessible and amenable.

We open this issue with an exclusive A Model for Life interview between Jeroen Bakkers and Didier Stainier (Stainier and Bakkers, 2023), in which they discuss, among other topics, the zebrafish model and its advantages and disadvantages. Both scientists are leading experts in using zebrafish to model heart development, disease and regeneration, and have actively contributed to the discovery of new genes and protein functions. In this interview, they frame the zebrafish model for heart research through the specific example of the *cloche* mutant, from initial discovery to the final exhaustive characterisation. One of the take-home messages from this interview is that there is still so much to learn from how the zebrafish regenerates its heart. Recent original research publications confirm this (Hu et al., 2022; Ogawa et al., 2021; Sun et al., 2022; Nguyen et al., 2023).

Model organisms like zebrafish have been instrumental in advancing this field, as they offer transparent embryos that are amenable to genetic manipulations and live imaging. In addition, the recent developments in *in vitro* models to study human development such as organoids (Drakhlis et al., 2021), gastruloids (Rossi et al., 2021), cardioids (Hofbauer et al., 2021) and epicardioids (Meier et al., 2023) will provide new research opportunities to study cardiac development and related diseases. These new *in vitro* modelling systems are summarised in the beautiful At a Glance poster article by Lika Drakhlis and Robert Zweigerdt, which clearly explains the different types of *in vitro* cardiac models, their nomenclature and the steps of mammalian cardiogenesis that these models attempt to recapitulate (Drakhlis and Zweigerdt, 2023). A key message is that every model comes with advantages and limitations: it is up to the researchers to (1) choose the best model(s) that fits the biological question, and (2) decide which (if any) of the aspects need to be optimised or improved for a specific research purpose.

The A Model for Life interview with Professor Christine Mummery offers an additional, more personal, perspective on the importance of advanced *in vitro* cardiac model systems in solving the challenges of heart failure and beyond (Mummery, 2023).

Despite their clear strengths, *in vitro* and embryonic zebrafish models cannot effectively recapitulate the complex circulation of the human heart. To model this process and the consequences of its perturbations, both large and small mammalian models of heart failure are invaluable. In this Special Issue, a paper by Cacheiro et al. (2023) reminds us that human genetic cardiomyopathy phenotypes are generally well recapitulated in genetically modified mice, in terms of both cardiac and systemic manifestations of genetic syndromes. As genomics research identifies more and more loci associated with human heart failure, resources such as the International Mouse Phenotyping Consortium can help to identify and model pathogenic mutations. Alongside zebrafish, mice and human stem cell-derived *in vitro* systems, articles in this issue also discuss the use of rats (Jiang et al., 2023; Zhang et al., 2023) and pigs (Salomon et al., 2023; Wolf et al., 2023) as valuable models with strong translational potential, expanding the array of models to investigate the causes, consequences and cures for heart failure.

## Congenital heart disease

During development, a complex interaction between cells, tissues and organs is required to form the embryo proper, and impairment of these interactions may result in congenital defects or even miscarriages. In this issue, Boezio et al. (2023) use zebrafish to study the role of the epicardium, the outermost layer of the heart, in coordinating growth of the myocardium. The authors used different genetic approaches to deplete epicardial cells from the developing heart and observed that, in early developmental stages, this results in reduced hypertrophic growth (increase in cell size), whereas at later developmental stages this results in reduced hyperplastic growth (more cells). One of the mechanisms that the authors identify as being responsible for these growth defects is the secretion of FGF and VEGF (Boezio et al., 2023). These results may be relevant to better understand the pathophysiology of complex congenital heart defects, such as hypoplastic left heart syndrome, in which growth of the left ventricle is severely impaired during development. Although defective growth of the myocardium is a hallmark of this disorder, the growth defects may be, in part, the consequence of impaired interactions with other cell types. For its proper development, the embryo depends on nutrition from the mother, either stored in the egg yolk or provided through the placenta. Sally Dunwoodie and colleagues study a metabolic disease caused by NAD deficiency, which results in congenital heart malformation, embryo growth retardation and miscarriage (Cuny et al., 2023). Their work, published in this issue of DMM, shows that embryos developing in dams carrying a heterozygous mutation in the *Slc6a19* gene, which encodes an amino acid transporter, and being fed a specific diet lacking tryptophan display a variety of congenital defects (Cuny et al., 2023). Interestingly, the severity of the phenotypes was independent of the genotype of the pups. The authors concluded that *Slc6a19* haploinsufficiency in the mother results in reduced tryptophan uptake that, when combined with a tryptophan-deprived diet, results in reduced NAD levels in the circulation with severe consequences for embryo development. This study is an excellent example of the complex gene–environment interaction that one needs to consider when studying complex disorders such as congenital heart malformations.

## Inherited cardiomyopathy

Inherited cardiomyopathies are an important cause of sudden death. Recent research highlights the importance of genetic testing and of accurate modelling to understand the genotype–phenotype relationship in a broad spectrum of cardiomyopathies.

Cotticelli et al. (2023) used human induced pluripotent stem cell (hiPSC)-derived cardiomyocytes to generate isogenic models of Friedreich's ataxia by silencing frataxin expression in the cardiomyocytes. The authors used an interesting approach to generate isogenic mutated and non-mutated cardiomyocytes, i.e. frataxin knockdown by small interfering RNA (siRNA) in wild-type hiPSC-derived cardiomyocytes (Cotticelli et al., 2023). This approach can be considered a complementary technique to the generation of isogenic hiPSC lines by gene editing followed by differentiation of the isogenic lines. The authors highlight the advantage of their approach as being less labour intensive than the generation of a stable gene-edited line. Although we agree that this might be true for a specific and limited number of experiments, we also argue that the choice of gene expression modulation approach should equally depend on the number of experiments required and on the underlying biological question. Also, much like hiPSC differentiation, RNA interference (RNAi) efficiency can vary across independent experiments. Nevertheless, we appreciate that RNAi is of particular value when studying mutations that possibly interfere with the expansion and differentiation of the iPSC line. In particular, triplet repeat expansions, which cause Friedreich's ataxia when they occur in the gene that encodes frataxin, are a type of mutation that is quite unstable in culture and can lead to genomic instability and difficulties in maintaining and differentiating hiPSCs. The authors of this study optimised RNAi-based silencing to achieve more consistent and robust transcriptional changes of genes important for mitochondrial function and inflammation. Interestingly, lack of frataxin also correlated with increased activation of the interferon response, probably in response to release of the mitochondrial DNA (Cotticelli et al., 2023). This work overall confirms the value and, importantly, versatility of hiPSC-derived cardiac disease models.

Genome-wide association studies have identified many single-nucleotide polymorphisms (SNPs) in non-coding DNA that increase the risk of developing a cardiac disease (Villar et al., 2020). The individual effects of these SNPs on disease outcome may be small, but they may increase when SNPs occur in combination with other genetic traits, so-called genetic modifiers. New tools and genetically tractable models are needed to address how these genetic traits interact to cause or affect cardiac diseases. To help address this challenge in the field, Ding et al. (2023) developed a F0 CRISPR-based genetic assay in an adult zebrafish cardiomyopathy model to screen for genetic modifier genes. By validating their approach in stable genetic mutants, the authors confirmed that their approach can identify novel genetic modifiers in adult cardiomyopathy (Ding et al., 2023). They recognise that the F0-based phenotypes can be variable and do not always recapitulate the disease traits seen in stable zebrafish mutants, which may be related to the incomplete mutagenesis and/or off-target effects, as also seen in other knockdown approaches. Altogether, their approach adds to the growing toolbox for studying clinically relevant genetic traits for cardiac diseases in zebrafish. The scientific rigour of this screen, innovative screening approach and high translational potential for the identification of human cardiac disease-related genetic modifiers has prompted us to select this particular article as this issue's Editors' choice. We congratulate the authors of this paper – Yonghe Ding, Mingmin Wang, Haisong Bu, Jiarong Li, Xueying Lin and Xiaolei Xu – on their excellent work.

## Acquired heart disease

Heart disease that sets the path towards heart failure can present after birth and during adulthood as a consequence of other conditions, such as coronary heart disease, infections, acute or chronic diseases that affect other organs than the heart, high blood pressure, valve disease and many others (Tsao et al., 2023).

Myocardial loss as a consequence of injury is largely irreversible. Translatable strategies to promote tissue regeneration or to mitigate tissue loss could fundamentally address myocardial deficits. Prior work showed that genetic deletion of Tip60 promotes functional recovery after experimental myocardial infarction with evidence of cell cycling (Wang et al., 2022). Building on this, the same group published a follow-up study in this issue of DMM demonstrating that a pharmacological inhibitor of Tip60, TH1834, can reproduce some of the favourable effects of genetic Tip60 deletion (Wang et al., 2023). To mitigate cardiac remodelling upon injury, Todorova et al. (2023) show that the U.S. Food and Drug Administration (FDA)-approved drugs AMD3100 and the B3-AR agonist mirabegron are associated with the mobilisation of mesenchymal stem cells and with markers of improved cardiac function when administered to rats after experimental myocardial infarction. Interestingly, these effects were also associated with increased vasculature within the border zone (Todorova et al., 2023), providing a potential clue for another mechanism to prevent myocardial loss and, consequently, progressive heart failure. Alongside studies investigating intrinsic biological processes, researchers are also exploring cell- and tissue-based interventions to regenerate the injured myocardium. In this issue, Florian Weinberger's group describe the use of immature engineered heart tissue patches to repair myocardial injury (von Bibra et al., 2023).

DUSP6 is a protein that has already been investigated in the context of heart regeneration in zebrafish, mice and rats. In their new article, Zhang et al. (2023) move their focus to the inflammatory cells that are normally activated upon cardiac injury: monocytes and macrophages. They show how pharmacological inhibition of DUSP6 prevents abnormal cardiac remodelling upon experimental myocardial infarction by suppressing macrophages and inflammation. This work nicely underscores the role of macrophages in heart physiology and disease that is increasingly attracting attention (Huang et al., 2020; Hulsmans et al., 2017). Regulating the inflammatory and reparative response after myocardial infarction is a delicate balance – but an attractive therapy to improve heart regeneration.

The generation and analysis of rats with cardiac-specific knockout of Trim44 by Jiang et al. (2023) demonstrated that this protein is important for heart growth under basal conditions, but that it is also an important mediator of cardiac hypertrophy induced by adrenergic stimulation. Their study corroborates the value of tissue-specific knockout approaches in animal models: it enables the identification of the role of a particular gene in the specific organ or tissue of interest. Indeed, Trim44 had already been associated with other diseases, in particular cancer (Xiao et al., 2020), but its function in the heart remained largely unknown. Knowledge of Trim44 involvement in the PIK3/AKT/mTOR signalling pathway enabled the authors to test and prove the hypothesis that adrenergic-induced cardiac remodelling is mediated by Trim44 through the same signalling cascade.

These type of models and approaches are valuable in providing a better understanding of the complex network underlying physiological heart development and growth and its pathological remodelling, and provide a rationale for mechanistic intervention development.

Finally, sometimes the treatment of heart failure, including corrective surgeries with the need for cardiopulmonary bypass, can lead to terminal inflammation. These processes are the topic of intense investigation, because reducing the unwanted side effects can significantly improve patient outcomes. Salomon et al. (2023) use a piglet model of cardiopulmonary bypass to suggest that cardiac pulmonary bypass can disrupt the microbiome. Interestingly, a complementary study by Naghipour and colleagues investigates how the gut microbiome metabolite trimethylamine-N-oxide affects heart function in the context of cardiovascular disease (Naghipour et al., 2023). Taken together, these two studies emphasise the complex roles of the gut microbiome in maintaining heart health and exacerbating disease. This knowledge could inform complementary therapies to reduce terminal inflammation and other complications of current heart disease and failure treatment strategies.

Despite significant progress in understanding and treating the underlying causes of heart failure, organ transplantation remains a keystone therapeutic option, but is significantly hampered by severe donor organ shortages. Xenotransplatation has long been a faraway possibility, but, as Eckhard Wolf and colleagues discuss in their Perspective, the community is inching closer to a realistic prospect (Wolf et al., 2023).

## Conclusions

The Research, Resource and Review-type articles in this Special Issue cover a wealth of seemingly diverse topics. However, preventing, understanding and treating heart failure are the common threads that bind these articles. Importantly, these articles are openly accessible to all interested researchers, patients and clinicians who work tirelessly to advance the field. Better models, refined analytics, richer patient datasets and innovative therapeutic interventions are already moving heart failure towards clinical success. We look forward to future research advances that will undoubtedly enrich DMM's ongoing subject collection and the field as a whole.
